# Frequency of Magnetic Resonance Imaging patterns of tuberculous spondylitis in a public sector hospital

**DOI:** 10.12669/pjms.321.8524

**Published:** 2016

**Authors:** Sumera Tabassum, Shahbaz Haider

**Affiliations:** 1 Dr. Sumera Tabassum, MBBS, FCPS, Consultant Radiologist, Department of Radiology, Jinnah Post Graduate Medical Centre (JPMC), Karachi, Pakistan; 2 Dr. Shahbaz Haider, MBBS, FCPS, Senior Registrar, Medical Unit IV, Ward 23, Jinnah Post Graduate Medical Centre (JPMC), Karachi, Pakistan

**Keywords:** Tuberculous, Spondylitis, Magnetic resonance imaging

## Abstract

**Objective:**

To determine frequencies of different MRI patterns of tuberculous spondylitisin a public sector hospital in Karachi.

**Methods:**

This descriptive multidisciplinary case series study was done from October 25, 2011 to May 28, 2012 in Radiology Department and Department of Medicine in the Jinnah Postgraduate Medical Center Karachi. MRI scans (dorsal / lumbosacral spine) of the Patients presenting with backache in Medical OPD, were performed in Radiology Department. Axial and sagittal images of T1 weighted, T2 weighted and STIR sequences of the affected region were taken. A total of 140 patients who were diagnosed as having tuberculous spondylitis were further evaluated and analyzed for having different patterns of involvement of the spine and compared with similar studies.

**Results:**

Among frequencies of different MRI pattern of tuberculous spondylitis, contiguous vertebral involvement was 100%, discal involvement 98.6%, paravertebral abscess 92.1% cases, epidural abscess 91.4%, spinal cord / thecal sac compression 89.3%, vertebral collapse 72.9%, gibbus deformity 42.9% and psoas abscess 36.4%.

**Conclusion:**

Contiguous vertebral involvement was commonest MRI pattern, followed by disk involvement, paravertebral & epidural abscesses, thecal sac compression and vertebral collapse.

## INTRODUCTION

Tuberculosis is a chronic inflammatory granulomatous disease caused by Mycobacterium tuberculosis.^1^ One third of the world’s population is infected with Mycobacterium tuberculosis. Spinal Tuberculosis, also called tuberculous spondylitis, makes 50% of the bony cases and 0.5 -1.5% of all cases of tuberculosis.^2^,^3^ Clinical symptoms of tuberculous spondylitis i.e. Fever, malaise, backache and focal tenderness are often insidious. Diagnosis of infection of the spineby M. tuberculosis requires high index of suspicion.^1^

Ability of Magnetic Resonance Imaging (MRI) to detect disc, end plate, vertebral and soft tissue changes are unique.^4^ Ithas allowed us to diagnose Spinal Tuberculosis in less advance stage.^5^ A combination of well defined paraspinal abnormal signal and a thin and smooth abscess wall is seen in 90%of tuberculous spondylitis and zero % in pyogenic spondylitis. In addition features like presence of paraspinal or intraosseous absess, subligamentous spread or more than three vertebra makes the reviewer/radiologist identified sensitivity, specificity and accuracy value of diagnosis of Tb spondylitis100%, 80%, and 90%, respectively.^6^,^7^

Our objective was to find the frequencies of the different magnetic resonance imaging patterns of tuberculous spondylitis in general public.

## METHODS

This descriptive multidisciplinary case series study was done from October 25, 2011 to May 28, 2012 in Radiology Department and Department of Medicine in the Jinnah Postgraduate Medical Center Karachi.

Both male and female patients with age between 15 to 40 years having backache of dorsal or lumbar region for more than three months coming to Medical OPD of JPMC were advised to have MRI scan of relevant region (dorsal or lumber) from Radiology Department JPMC. Patients who were diagnosed as having tuberculous spondylitis after MRI scan and were willing to give informed consent were further evaluated for MRI patterns.

Patients having history of trauma to spine, neck pain, having pain of less than three months duration, and patients with MRI finding other than tuberculous spondylitis e.g. prolapsed disc or metastasis were excluded. Similarly patients having previous spinal surgery, having any MRI-incompatible device in body and refusing to give consent were also excluded from the study.

The study was conducted after the approval of ethical committee. All the patients meeting criteria were included in this study. The MRI scan of thoracic or lumbar spine of the selected patients were performed on ARCHIEVA NOVA DUAL PHILIPS 1.5 TESLA MRI machine by a trained MRI technician having more than three years experience. Axial and sagittal images of T1 weighted, T2W and STIR sequences of the affected region were taken. MRI scan of thoracic or lumbar spine of the patients after being diagnosed as having features of Tuberculous spondylitis after MRI scan were further evaluated and analyzed for having different patterns of involvement of the spine.

In this study diagnosis of Spinal tuberculosis and its differentiation from pyogenic was based on studies by Jung^6^ and Smith.^7^ With help of features described,^6^,^7^ diagnosis of tuberculous spondylitiswas made with sensitivity, specificity, and accuracy values of 100%, 80%, and 90%. Similarly features described in favour of pyogenic spondylitis, were used by the radiologists for exclusion of pyogenic cases from study with sensitivity, specificity, and accuracy values of 80%, 100%, and 90%.^6^,^7^ Images were reviewed by two senior radiologists having three years post fellowship experience.

Data was entered and analyzed on SPSS 10.0 software. Frequencies and percentage were computed for gender, age, region of involvement, presence of fever and different patterns of tuberculous spondylitis. Mean, standard deviation and 95% confidence interval were computed for age and duration of back ache.

## RESULTS

A total of 140 patients having backache were found to have spinal tuberculosis. The average age of the patients was 30.17±7.13 years (95%CI: 28.98 to 31.36). Similarly the mean duration of back ache of the patients was 6.20±2.04 months. Fever was observed in 40.7% (n=57) cases and history of tuberculosis was reported in 36.4% cases. Out of 140 cases, 57(40.7%) were male and 83(59.3%) were female. In 77(55%)region involved was thoracic region and in 45% (n= 63) Lumber region. Vertebra most commonly involved was first Lumber (L1 in;30.8% n=43)followed by second lumber (L 2 in; 27.1% (n=38). L1 was involved in 12.9% (n=18) in combination with second lumber, in 10.0% (n=14) in combination with 12th thoracic vertebra and in 7.9% (n= 11)cases in combination with both T12 and L1 vertebrae.

Frequencies of different magnetic resonance imaging pattern of tuberculous spondylitis were contagious vertebrae involvement in 100% cases (n= 140). Two contagious vertebrae were involved in 76.4% cases (n=107), while more than two in 23.6% (n=33). Frequencies among three, four and five contagious vertebrae involvement, i.e. Frequencies among ‘more than two vertebrae’ category were, 20% (n=28), 2.9% (n=4) and 0.7% (n=1) respectively.

Discal involvement was seen in 98.6%. Abscesses were seen in 93.57% cases, among these paravertebral abscess in 92.1% cases, epidural abscess in 91.4%, psoas abscess was seen in 36.4% cases, more than one type seen together.

Spinal cord / thecal sac compression in 89.3%, vertebral collapse in 72.9% and gibbus deformity in 42.9%. Comparison of different MRI findings in male and female gender is given in Table-I. Frequency of vertebral collapse was significantly high in patients below 30 years of age than above 30 years of age.

**Table-I:** Frequency of different magnetic resonance imaging pattern of tuberculous spondylitis with respect to gender (n=140).

Different Magnetic Resonance Imaging Pattern	Male n=57	Female n=83
Discal Involvement	57(100%)	81(97.6%)
Vertebral Collapse	36(63.2%)	66(79.5%)
Epidural Abscess	53(93%)	75(90.4%)
Spinal Cord/Thecal Compression	55(96.5%)	70(84.2%)
Paravertebral Abscess	52(91.2%)	77(92.8%)
Gibbus Deformity	28(49.1%)	32(38.6%)
Psoas Abscess	28(49.1%)	23(27.7%)

## DISSCUSSION

This study on tuberculous spondylitis showed female preponderance (59.3% vs 40.7% for male). We included the patients of age range of 15-40 years. In this age range mean age was 30.71 years. Among two regions, thoracic and lumber, included in study, thoracic vertebrae were involved in 55% (77/140) cases while in rest (45%) lumber vertebrae were involved. Vertebra most commonly involved was first Lumber (L1 in;30.8% n=43)followed by second lumber (L 2 in; 27.1% (n=38). Two contagious vertebra were involved in 76.4% cases (n=107), while three, four and five contagious vertebrae were involved in 20% (n=28), 2.9% (n=4) and 0.7% (n=1) cases respectively.

Among different MRI patterns of tuberculous spondylitis, disc involvement, paravertrebral abscess and epidural abscess were the commonest (98.6%, 92.1% and 91.4% respectively). Spinal cord/thecal sac compression was observed in 89.3%. Gibbus deformity was seen 42.9% and was the marker of advanced disease. Psoas abscess is unique due to Psoas muscle’s anatomical attachments to lumber vertebrae and femur^8^ as abscess arises as paravertebral abscess but usually extends down into the thigh and therefore is reported separately. Psoas abscess was observed in 36.4% cases. Combining all patients with any of the abscesses, Abscess formation was observed in 93.57% of the patients including 92.1% with paravertebral, 91.4% with epidural abscess and 36.4% with psoas abscess; paravertebral and epidural abscess mostly in combination. Frequency of psoas abscess in cases where tuberculosis involves lumbar vertebrae came to be 80.8%.

Although many studies have been done on tuberculous infection of spine in Pakistan, but there is scarcity of studies elaborating the MRI findings of tuberculous spine. In one study by Nasreen Naz, Lutfullah Balochii^9^ in 2007, most specific investigatin used was plain radiograph of the spine. In another study in 2008 by Nasreen Naz, Aslam Siddiqui,^10^ regarding imaging, plain radiography was relied for diagnosis of spinal tuberculosis.

In study by Chandir^11^ Spinal tuberculosis was shown to be 26.3% among extrapulmonary Tuberculosis but MRI was not involved in diagnostic process. In a study by Ahmed N^12^ in 2004, MRI modality was used for spine, but focus was to differentiate between different causes like neoplastic, infective and non compressive mylopathy as cause of Spinal cord disease. No particular focus on tuberculous spine was done. In a study by Zaidi^13^ in 2010, details of tuberculous involvement of spine were well worked up.

In our study there was female preponderance (59.3% vs 40.7%). Among the patients having spinal tuberculosis high female preponderance was also observed in national and international studies.^13^,^14^ In study by Zaidi H^13^ conducted in Military Hospital female patients were 52%. Comparatively lesser percentage may be explained by the fact that military hospitals mainly serve the male dominant population as many of the families of military employees reside in areas away from posting places of employees. Preponderance of female gender in studies of our region may be explained by the fact that female are comparatively more deprived. Some studies have showed male preponderance instead.^15^,^16^ Mean age in study by Moon^17^ was 38 years. Lower mean age in our study (30.71 years) was due to the fact that we included the patients of age range of 15-40 years only. Among the thoracic and lumber regions included in our study, thoracic vertebrae were involved in 55% (77/140) cases while in rest (45%) lumber vertebrae were involved. In literature Thoracic spine is frequently reported as the most common site of involvement in spinal tuberculosis^18^-^20^ followed by lumbar and cervical spines. This is contrary to the findings of Sinan^21^ where involvement was greater in the lumbar spine. Thoracolumbar junction has been described the most common single site of involvement in some studies.^22^,^23^ Rasit^24^ reported the 9^th^ thoracic vertebra as the most common seen to be involved. In our study Vertebra most commonly involved was first Lumber (L1 in;30.8% n=43) followed by second lumber (L2 in; 27.1% (n=38). In literature more than one vertebral body is commonly involved,^21^,^25^ and involvement of vertebral bodies is usually contiguous.^2^ In our study two contagious vertebra were involved in 76.4% cases (n=107), while three, four and five contagious vertebrae were involved in 20% (n=28), 2.9% (n=4) and 0.7% (n=1) cases respectively.

In Alothman’s study,^26^ 80% of patients had paraverebral abscess, Omari,^27^ had also detected psoas and paravertebral abscesses in 10 out of 11 patients with tuberculosis spondylitis indicating high prevalence rate of abscess formation in this disease.

Involvement of two contiguous vertebrae was seen in 100 percent cases and so most frequent pattern. In Study by Zaidi^13^ although most frequent pattern described was contiguous vertebral involvement but percentage given was 65.3%which was lesser than other frequencies given in same study^13^ i.e. vertebral body involvement (100%), disc involvement (77.3%) and similarly less than spinal cord compression & epidural abscess in study by Zaidi.

In our study next most frequent pattern was disc involvement (98.6%). It shows the rare possibility of absence of disc involvement in 1.4%cases in the presence of involved vertebrae on both sides of the uninvolved disc and may be by spread through venous system or along soft tissue planes.^2^ Frequency of paravertebral abscesses was also veryhigh (92%). Similar high frequency was also reported by Zaidi.^13^

Spinal cord / thecal sac compression was observed in our study 89.3%. Frequency of disc involvement (98.66%) and frequency of Spinal cord/thecal sac compression in our study (89.3%) were higher as compared to study by Zaidi, in which disc involvement and spinal cord/thecal sac compression were 77.3% cases and 64% cases respectively.

It points towards the possibility of early imaging by Magnetic resonance radiography in Military population by Military Hospital as compared to that in general public Hospital’s served population and may reflect earlier detection of disease in Military Hospital served population. It also supports the earlier need for advising MR imaging in patients having the complaints of vertebral column in countries where tuberculosis is quite common. Exclusion of tuberculous spondylitis by MRI scanning is also very important as it provides relief to patient and more so to treating physician.

In this study diagnosis of Spinal tuberculosis and its differentiation from pyogenic, as pointed in the introduction and results, was based on studies^6^,^7^ showing that a combination of well defined paraspinal abnormal signal and a thin and smooth abscess wall is seen in 90% of tuberculous spondylitis and 0% in pyogenic spondylitis. In addition features like presence of paraspinal or intraosseous absess, subligamentous spread or more than 3 vertebra supports / strengthens the diagnosis of Tuberculous spondylitis. Knowledge and use of all these features by the radiologist, diagnosis of tuberculous spondylitis is made with sensitivity, specificity, and accuracy values of 100%, 80%, and 90%, respectively. Features in favour of pyogenic spondylitis has been determined to be an ill-defined paraspinal abnormal signal, absence of paraspinal or intraosseous abscess, subligamentous spread to fewer than three vertebral levels or without subligamentous spread, a thick and irregular abscess wall, a horizontal band like sparing of the body, and involvement of two or fewer vertebral bodies. Seeing these finding which are differentiating features for pyogenic spondylitis from tuberculous spondylitis, the radiology diagnosis of pyogenic spondylitis can be made with sensitivity, specificity, and accuracy values of were 80%, 100%, and 90%.^6^

### Limitations of the study

As this study was done in public sector hospital of which most patients are poor and had low level of affordability, so the MRI studies with contrast agent were not performed. Similarly poor detection of calcification by MRI studies is another limitation and needs to be kept in mind while doing and analyzing studies involving MRI modality. Furthermore due to the same reasons narrated above, MRI scan was done only of the area/region complained of, and therefore involvement of the non contiguous vertebral and total number of vertebrae involved would not have been reported as infection would have been present in region other than the scanned one.

## CONCLUSION

Contiguous vertebral involvement was commonest MR imaging pattern, followed by disk involvement, paravertebral & epidural abscesses, thecal sac\spinal cord compression and vertebral collapse.
